# Supplementary material for: Empirical methods for the validation of time-to-event mathematical models taking into account uncertainty and variability: application to EGFR + lung adenocarcinoma
Source: BMC Bioinformatics. 2023 Sep 4;24:331. doi: 10.1186/s12859-023-05430-w (PMC10478282; doi:10.1186/s12859-023-05430-w)

**Supplementary Figures**

**Figure S1**

**Evolution of the ratio of non-significant bootstrapped log-rank tests on the entire population according to the number of bootstrap iterations.** The ratio can be considered as stable after 5000 iterations.


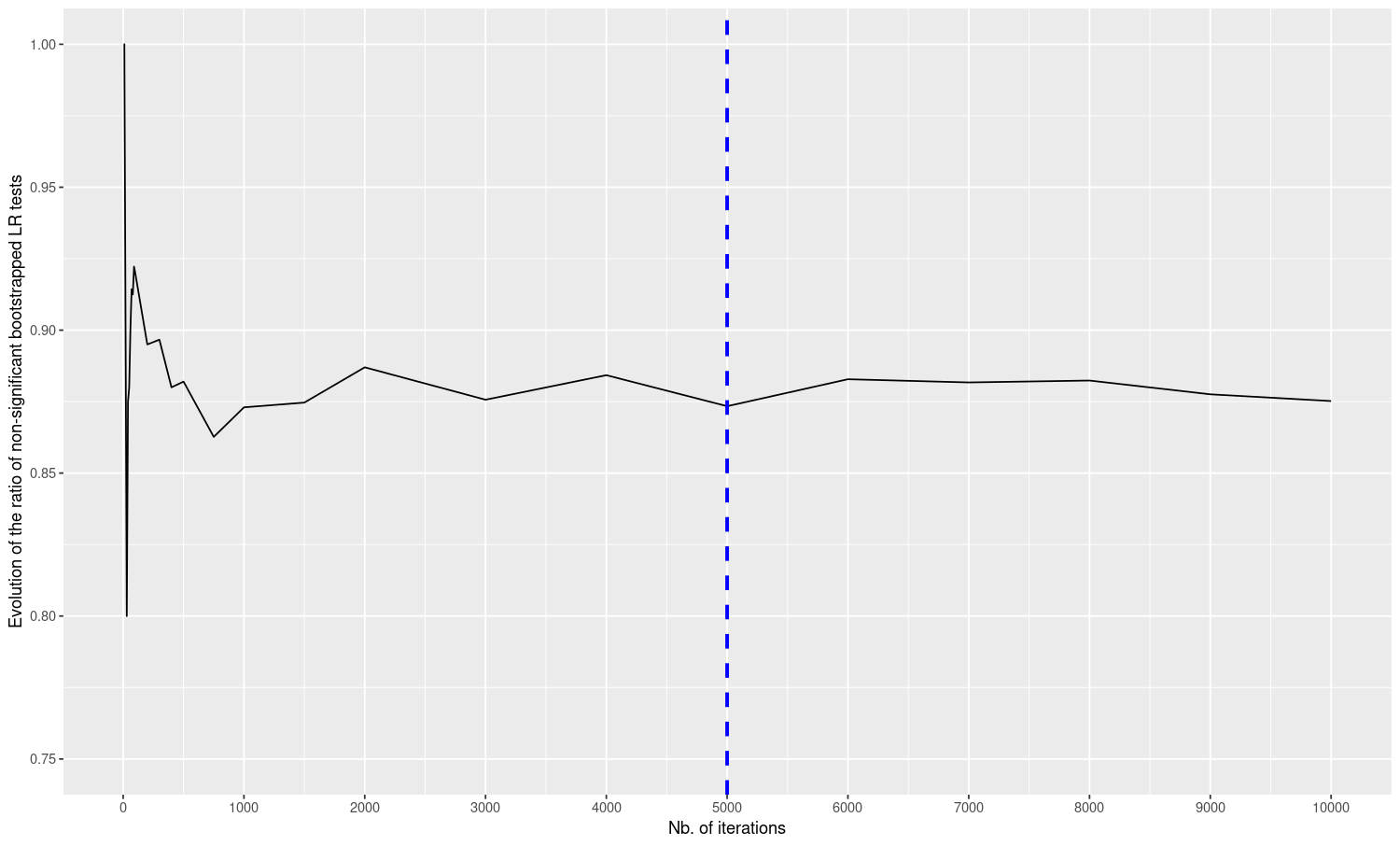

Supplement: Supplementary file 1 — Additional file 1: Evolution of the ratio of non-significant bootstrapped log-rank tests on the entire population according to the number of bootstrap iterations. Description: Display of the stabilization of a bootstrapped statistical test as the number of iterations increases demonstrating why 5000 iterations were considered as satisfactory. [file 12859_2023_5430_MOESM1_ESM.docx]
